# ImmunoTar—integrative prioritization of cell surface targets for cancer immunotherapy

**DOI:** 10.1093/bioinformatics/btaf060

**Published:** 2025-02-11

**Authors:** Rawan Shraim, Brian Mooney, Karina L Conkrite, Amber K Hamilton, Gregg B Morin, Poul H Sorensen, John M Maris, Sharon J Diskin, Ahmet Sacan

**Affiliations:** Division of Oncology and Center for Childhood Cancer Research, Children’s Hospital of Philadelphia, Philadelphia, PA 19104, United States; School of Biomedical Engineering, Science and Health System, Drexel University, Philadelphia, PA 19104, United States; Department of Molecular Oncology, BC Cancer Research Institute, Vancouver, BC V5Z 0B4, Canada; Canada’s Michael Smith Genome Sciences Centre, BC Cancer Research Institute, Vancouver, BC V5Z 4S6, Canada; Division of Oncology and Center for Childhood Cancer Research, Children’s Hospital of Philadelphia, Philadelphia, PA 19104, United States; Division of Oncology and Center for Childhood Cancer Research, Children’s Hospital of Philadelphia, Philadelphia, PA 19104, United States; Canada’s Michael Smith Genome Sciences Centre, BC Cancer Research Institute, Vancouver, BC V5Z 4S6, Canada; Department of Medical Genetics, University of British Columbia, Vancouver, BC V6T 1Z4, Canada; Department of Molecular Oncology, BC Cancer Research Institute, Vancouver, BC V5Z 0B4, Canada; Department of Pathology and Laboratory Medicine, University of British Columbia, Vancouver, BC V6T 1Z4, Canada; Division of Oncology and Center for Childhood Cancer Research, Children’s Hospital of Philadelphia, Philadelphia, PA 19104, United States; Department of Pediatrics, Perelman School of Medicine, University of Pennsylvania, Philadelphia, PA 19104, United States; Abramson Family Cancer Research Institute, Perelman School of Medicine, University of Pennsylvania, Philadelphia, PA 19104, United States; Division of Oncology and Center for Childhood Cancer Research, Children’s Hospital of Philadelphia, Philadelphia, PA 19104, United States; Department of Pediatrics, Perelman School of Medicine, University of Pennsylvania, Philadelphia, PA 19104, United States; Abramson Family Cancer Research Institute, Perelman School of Medicine, University of Pennsylvania, Philadelphia, PA 19104, United States; School of Biomedical Engineering, Science and Health System, Drexel University, Philadelphia, PA 19104, United States

## Abstract

**Motivation:**

Cancer remains a leading cause of mortality globally. Recent improvements in survival have been facilitated by the development of targeted and less toxic immunotherapies, such as chimeric antigen receptor (CAR)-T cells and antibody-drug conjugates (ADCs). These therapies, effective in treating both pediatric and adult patients with solid and hematological malignancies, rely on the identification of cancer-specific surface protein targets. While technologies like RNA sequencing and proteomics exist to survey these targets, identifying optimal targets for immunotherapies remains a challenge in the field.

**Results:**

To address this challenge, we developed ImmunoTar, a novel computational tool designed to systematically prioritize candidate immunotherapeutic targets. ImmunoTar integrates user-provided RNA-sequencing or proteomics data with quantitative features from multiple public databases, selected based on predefined criteria, to generate a score representing the gene’s suitability as an immunotherapeutic target. We validated ImmunoTar using three distinct cancer datasets, demonstrating its effectiveness in identifying both known and novel targets across various cancer phenotypes. By compiling diverse data into a unified platform, ImmunoTar enables comprehensive evaluation of surface proteins, streamlining target identification and empowering researchers to efficiently allocate resources, thereby accelerating the development of effective cancer immunotherapies.

**Availability and implementation:**

Code and data to run and test ImmunoTar are available at https://github.com/sacanlab/immunotar.

## 1 Introduction

Targeted and less toxic cancer therapies have revolutionized cancer treatment in the past decade (Zhang and Zhang 2020). Immunotherapy specifically has revolutionized cancer treatment by leveraging an individual’s immune system to combat cancer cells through natural mechanisms that are impeded during disease progression (Shahid *et al.* 2019, Peters *et al.* 2021). Several types of immunotherapies have shown success in cancer treatments such as immune checkpoint inhibitors (ICIs), which have shown remarkable effectiveness in tumors with a high mutational burden such as melanoma and lung cancers; however, ICIs remain ineffective in most low mutational burden cancers such as childhood malignancies (Long *et al.* 2022). Immunotherapies that engage T-lymphocytes, including chimeric antigen receptor (CAR) T-cell therapy, on the other hand, have shown notable efficacy and durable responses in hematological cancers for both pediatric and adult patients and are beginning to show signs of efficacy in solid tumors (Waldman *et al.* 2020, Haslauer *et al.* 2021, Fu *et al.* 2022). Antibody drug conjugates (ADCs), a type of monoclonal antibody immunotherapy that delivers cytotoxic drugs directly to the tumor, have proven efficacy in hematological tumors and in solid tumors, with a few already gaining FDA approval and many more in the clinical development pipeline (Fu *et al.* 2022).

Development of successful immunotherapies such as CAR-T cell therapy and ADCs involve identifying cancer-specific proteins to target. These proteins are traditionally surface proteins that arise from the accumulation of genetic or epigenetic aberrations which lead to expression of proteins that are either not found on normal tissue, or are present at elevated levels on tumor cells and very low levels on healthy cells, or are only present on germ and tumor cells (Abbott *et al.* 2020, Leko and Rosenberg 2020, Waldman *et al.* 2020). Technologies such as RNA-sequencing and mass spectrometry have been employed to explore the landscape of cell surface protein expression of cancers to identify these tumor-specific proteins (Hu *et al.* 2021, Ferguson *et al.* 2022, Mooney *et al.* 2023,  Hamilton *et al.* 2024).

Several criteria exist for an ideal surface protein immunotherapeutic target including high and homogeneous expression in cancer, minimal expression in normal healthy tissues, robust confidence in surface protein localization and functional relevance to the tumor (Wei *et al.* 2019, Abbott *et al.* 2020). Protein glycosylation is another factor to consider when selecting targets, as glycosylation patterns can be altered in cancer cells, leading to the presentation of cancer-specific epitopes (Ho *et al.* 2016). While publicly available databases allow investigation of these “ideal” surface protein characteristics, no tool currently enables users to query multiple databases simultaneously and systematically evaluate a protein's suitability as an immunotherapeutic target.

To address the urgent need to identify surface protein targets for therapeutic development, we developed ImmunoTar, a tool designed to handle user-provided cancer RNA or proteomic expression datasets, query each cancer protein across multiple public databases that tackle some of the criteria for an “ideal” therapeutic protein, and produce a score that quantitatively evaluates each protein as a potential immunotherapeutic candidate.

## 2 Materials and methods

**Figure 1.**
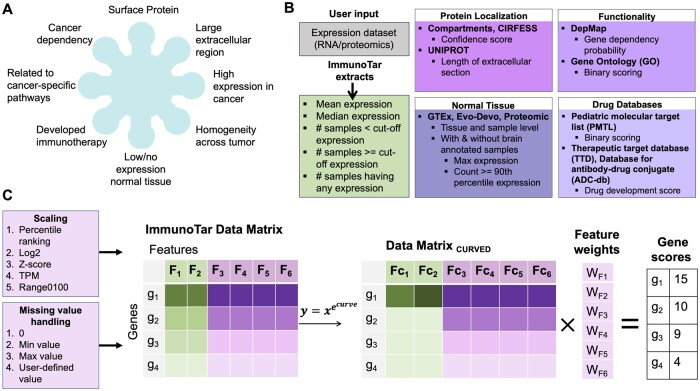
ImmunoTar feature extraction and surface protein scoring scheme. (A) The pre-defined criteria for an ideal immunotherapeutic target. (B) Summary of features extracted from the user-input expression dataset and the publicly available databases incorporated in ImmunoTar. (C) The steps of analysis within ImmunoTar. The first step involves extracting quantitative features from the user input and handle missing values of the extracted features using the methods listed. After rescaling and handling missing values of the features, the user has the option of applying a curving function to the feature values. After curving the feature values, a weight parameter can be applied and the final score of each gene is calculated as the sum of the weighted-curved-features.

ImmunoTar is a tool developed in the R programming language and is designed to integrate user-provided proteomic or RNA-sequencing expression datasets with publicly available databases. The goal of ImmunoTar is to assign scores to proteins representing their predicted suitability as an immunotherapeutic target. We equipped ImmunoTar with both adult-derived and pediatric-specific databases to aid the development of immunotherapy in childhood cancers as well as adult cancers. The databases included within ImmunoTar are selected to tackle the ideal immunotherapeutic target criteria and can be divided into four categories: normal tissue expression, protein localization, biological annotation, and reagent/therapeutic availability (Fig. 1A; Supplementary Table S1). For normal tissue expression we included adult-derived RNA-sequencing data from the Genotype-Tissue Expression (GTEx) and pediatric-derived RNA-sequencing data from the Evo-Devo Mammalian organs (Evo-Devo) project (Consortium 2013, Cardoso-Moreira *et al.* 2019). Acknowledging potential disparities between RNA expression and protein abundance, we also incorporated proteomics data derived from adult-normal human tissues generated by Jiang and colleagues (Jiang *et al.* 2020). For protein localization information, we included data from Compiled Interactive Resource for Extracellular and Surface Studies (CIRFESS), COMPARTMENTS, and UniProt (Apweiler *et al.* 2004, Binder *et al.* 2014, Waas *et al.* 2020). To gain deeper insights into the biological roles of protein targets, data from the DepMap project and Gene Ontology (GO) were added (Ashburner *et al.* 2000, Dwane *et al.* 2021). Identifying novel immunotherapeutic targets is inherently challenging due to the high possibility of poor or non-existent reagents, as highlighted in other studies (Mooney *et al.* 2023). To address this, we have incorporated data from the Therapeutic Target Database (TTD), the Database of Antibody-drug Conjugates (ADCdb), and the National Cancer Institute (NCI) relevant Pediatric Molecular Targets List (PMTL, https://moleculartargets.ccdi.cancer.gov/mtp-pmtl-docs), which helps users determine if the targets they are interested in have been identified and validated in other disease phenotypes (Chen *et al.* 2002, Shen *et al.* 2024). This integration facilitates the prioritization of targets with established reagents, enhancing the potential for successful validation and development.

ImmunoTar analyzes a dataset (project) in three main steps: (i) Generating a gene-by-features data matrix, (ii) Applying project analysis parameters to the data-matrix, and (iii) Calculating the ImmunoTar gene score and evaluating how the algorithm performed in prioritizing targets. Project analysis parameters are customizable as ImmunoTar is designed to accept user-input for the parameters using a project parameters file (Supplementary File S1).

Step one of the analyses involves extracting summary features for each gene in the expression dataset that is provided by the user and enriching with quantitative features from the public databases that are incorporated within ImmunoTar (Fig. 1B; Supplementary Table S2). The output from step one is a genes-by-features data-matrix (Fig. 1C). The user has the flexibility to select the summarization and enrichment database features that they desire to include in the genes-by-features data-matrix within the project analysis parameters file, with the default being to include all.

In step two, ImmunoTar applies additional project analysis parameters to the genes-by-features data-matrix. Initially, ImmunoTar will rescale and fill in any missing values for all features in the data-matrix (Fig. 1C). Next, ImmunoTar applies a curving parameter as a form of a non-linear normalization of the feature values. Feature-specific weights are then applied to produce the final feature value. The user can customize the parameter values associated with each of the features using the project analysis parameters file. Assigning different weights, curving, and scaling of feature values ensures that some features contribute more significantly to the final score of the gene in comparison to others.

**Figure 2.**
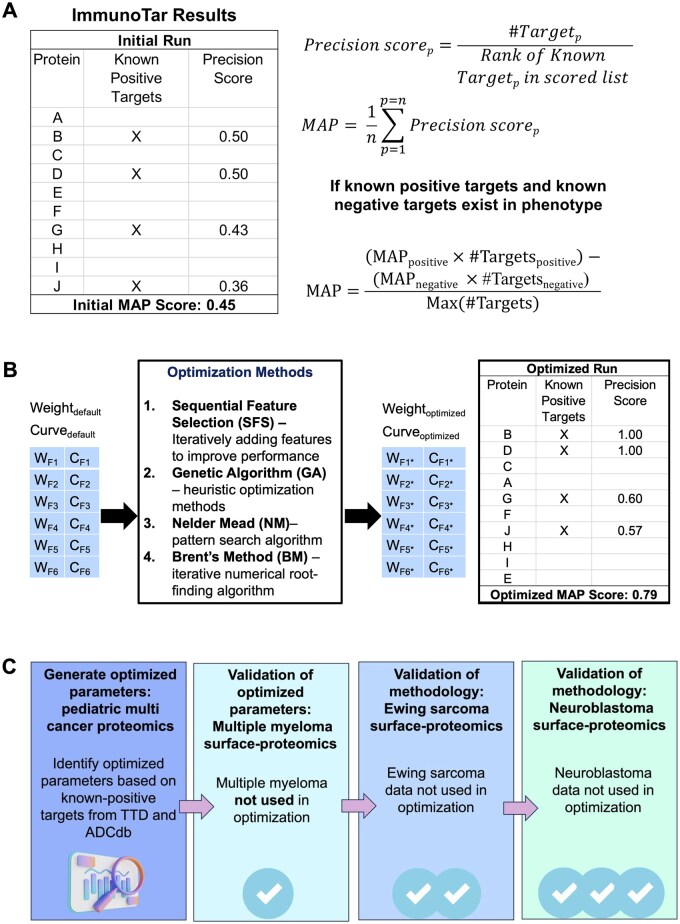
ImmunoTar evaluation, optimization, and validation datasets. (A) The mean average precision (MAP) score is used to evaluate the performance of ImmunoTar. The MAP score depends on the known-positive and known-negative targets assigned to each phenotype and is calculated based on the ranking of those targets after running ImmunoTar. (B) Optimization methods listed can be used to change the weights and curves of features. The goal of the optimization is to rank the known-positive targets higher in the results thus increasing the overall MAP score of the algorithm. (C) Optimized parameters are generated using a multi-cancer proteomic dataset and applied to other datasets to validate the approach. Validation datasets include multiple myeloma surface proteomics, Ewing sarcoma surface proteomics, and neuroblastoma surface proteomics.

The last step in the analysis is calculating the score for each gene using the weighted average of the final feature values (Fig. 1C). One of the outputs from ImmunoTar is a table that includes all of the compiled feature values and the final score assigned to each gene. To evaluate ImmunoTar’s performance in prioritizing targets, the mean average precision score (MAP) is calculated and given as an output with the analysis (Fig. 2A). The MAP score indicates how effectively immunotherapy targets in the queried cancer phenotype that are either in-clinic or in-development (known-positive targets) are ranked within ImmunoTar. The user can designate the known-positive targets in the project analysis parameters file or alternatively, they can be extracted using the therapeutic drug databases incorporated within ImmunoTar. If the user extracts this information using the available drug-databases, targets that have drugs that have been discontinued will be considered “known-negatives” and the MAP score will be calculated with those targets in consideration (Fig. 2A).

The curve and weight values influence the final feature values and thus the gene score and the MAP score of the algorithm. Weights affect feature values linearly, while the curve parameter provides an additional adjustment that emphasizes or de-emphasizes values within a feature distribution, depending on the curvature direction. Applying a positive curve value emphasizes higher feature values, accentuating differences among the top-ranking targets (Supplementary Fig. S1B). In contrast, a negative curve value highlights lower feature values, which can be advantageous for features where lower values are preferred (Supplementary Fig. S1C).

The weight and curvature values can be assigned by users based on prior knowledge, though this can introduce subjectivity into the scoring process. To enhance the objectivity of these assignments, we have incorporated multiple optimization methods within ImmunoTar aiming to maximize the MAP score of the algorithm. The optimization methods incorporated in ImmunoTar include sequential forward selection (SFS), Genetic Algorithm (GA), Nelder-Mead, and Brent’s Method, with each method following different strategies for optimization (Fig. 2B). SFS and GA are more exploratory optimization methods that help to broadly search the parameter value. SFS is a supervised search method that iteratively adds features as a score improves by a specific threshold, by default in ImmunoTar this is 0.01% (Pudjihartono *et al.* 2022). This approach will help to identify the subset of features that contribute the most to the overall score of a target (Pudjihartono *et al.* 2022). GA is a stochastic optimization technique that mimics the natural selection process by iterating through a population of potential solutions, in this case, weight and curvature values, to identify the optimal combination of parameters that maximizes the MAP score (Aliani *et al.* 2024). This method is specifically used when exploring various values where multiple local optima can exist (Aliani *et al.* 2024). Nelder-Mead and Brent’s Method would be categorized as local search methods, generally applied after the exploratory methods to further refine the parameter values. Nelder-Mead is a derivative-free optimization technique that keeps track of a set of potential solutions represented as numerical vectors and repeatedly adjusts their positions toward the most promising solution (Nelder and Mead 1965, Soritz *et al.* 2022). Brent’s method is a non-linear algorithm used to find the local maximum by adjusting a single parameter at a time (Brent 2013, Soritz *et al.* 2022). Combining multiple optimization methods will ensure a robust exploration of parameter values and the refining of final values to increase the MAP score.

## 3 Results

**Figure 3.**
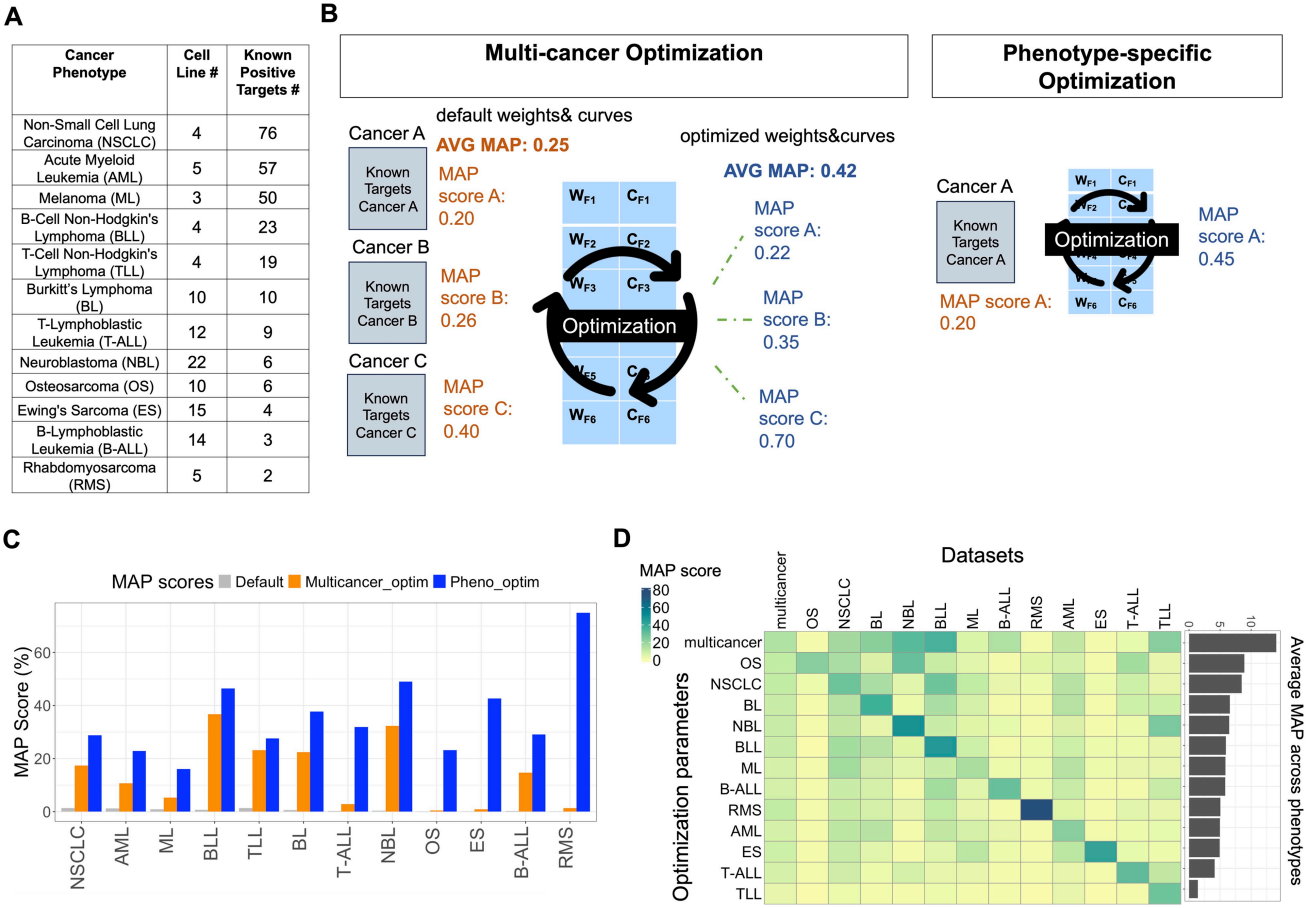
Optimization of ImmunoTar parameter values using pediatric pan-cancer expression data. (A) The pediatric cancer phenotypes included in the optimization scheme, listing the number of cell lines and the number of known-positive surface protein targets extracted from the drug databases TTD and ADCdb (Supplementary Table S2). (B) Optimization parameters were generated using two approaches, the first being a multi-cancer optimization approach and the second being a phenotype-specific optimization approach. (C) Using the multi-cancer optimization parameters, the MAP score for each individual phenotype increased at varying levels within phenotypes. The phenotype-specific optimization approach increased the MAP score further for each phenotype. (D) While the phenotype-specific parameters performed better on the phenotype itself, they did not perform as well on other phenotypes. Overall, the multi-cancer optimization parameters performed best across all phenotypes with an average MAP score of 14%.

Through this work, we assessed ImmunoTar and its analysis parameters using diverse cancer datasets (Fig. 2C). Step one of the analyses involved generating optimized analysis parameter values using a full proteome dataset that surveyed 12 pediatric cancer phenotypes with known-positive targets (Fig. 3A). We implemented two optimization strategies, including a multi-cancer optimization approach, where parameter values are evaluated across multiple cancer phenotypes at the same time and a phenotype-specific optimization approach, where parameter values are optimized using a single phenotype (Fig. 3B). During this process, we assessed the analysis parameter values by comparing the MAP scores of the algorithm when using the default versus the optimized parameter values. The goal of this first step is to determine which optimization strategy is best to generate parameters and to provide parameters that users can utilize across various cancer phenotypes in ImmunoTar.

The second step of the analyses involved validating ImmunoTar’s methodology and the parameters generated in step one by testing on three different cancer datasets; surface proteomics data from Multiple Myeloma (MM) adult cell line models, surface proteomics data from Ewing Sarcoma (EwS) pediatric patient-derived-xenograft (PDX) and cell line-derived-xenograft (CDX) samples, and surface proteomics datasets for neuroblastoma (NBL) from pediatric PDX models (Ferguson *et al.* 2022, Goncalves *et al.* 2022, Mooney *et al.* 2023, Hamilton *et al.* 2024). The goal of this secondary analysis is to validate that applying ImmunoTar with optimized parameter values across various cancer phenotypes effectively identifies viable candidate targets for further validation.

Within cancer phenotypes, we compared the prioritized targets within ImmunoTar to those identified by other scoring workflows in MM, EwS, and NBL. This comparison involved ranking assessments and performing Gene-Set Enrichment Analysis (GSEA), testing the effectiveness of ImmunoTar in target selection and benchmarking it against existing strategies in the field. Finally, we analyzed the top targets identified by ImmunoTar in MM, EwS, and NBL datasets to confirm its potential in pinpointing viable immunotherapeutic targets, demonstrating its utility across different cancer types and data sources.

### 3.1 Optimized parameter values generated using a pediatric multi-cancer dataset increases ImmunoTar’s MAP score across cancer phenotypes

The curving and the weight parameter values are crucial in calculating the final gene score in ImmunoTar. To identify optimized curve and weight values per feature, we utilized proteomic data from cell lines representing twelve pediatric cancer phenotypes with known-positive targets (Jiang *et al.* 2020) (Fig. 3A). We first applied the default project analysis parameters to each of the phenotypes (Supplementary File S2). Known-positive cell surface therapeutic targets were extracted from TTD and ADCdb (Supplementary Table S3).

To generate the multi-cancer and phenotype-specific parameters, we applied a series of optimization methods in the following order. First to identify features contributing positively to the MAP score, we applied SFS combined with Brent’s optimization; a feature was included only if it improved the MAP score by 0.1%, and after inclusion, a search for the local maximum using Brent’s method was conducted (Chandrupatla 1997). The resulting optimization parameter values were then further refined using the genetic algorithm method, with a sub-optimization of the top-scoring parameters in the population using Nelder-Mead, only updating the weight or curve parameter values if it increased the MAP score by at least 0.1% (Scrucca 2013, Musafer *et al.* 2022).

In the multi-cancer optimization strategy, only 9 of the 40 total features were retained by the feature selection method to calculate the final gene score (Supplementary File S3). After multi-cancer optimization, the average MAP score across all twelve phenotypes increased significantly by a 27-fold change (*P*-value <.001) in comparison to the default parameters, with an increase in the MAP score observed for every phenotype at varying degrees (Fig. 3C). B cell non-Hodgkin’s Lymphoma and neuroblastoma exhibited the most significant increase in their MAP value post-optimization, reaching precision scores of 37% and 32%, respectively.

Our phenotype-specific optimization strategy increased the MAP score within each phenotype even further in comparison to the multi-cancer optimization strategy (Fig. 3C). However, the phenotype-specific optimization parameters did not perform as well as the multi-cancer optimization parameters when applied to other phenotypes. The average MAP score across phenotypes was the highest (14%) when using the multi-cancer optimization parameter values (Fig. 3D).

### 3.2 ImmunoTar validates ITGA4, ITGB7, and FLVCR1 as candidate immunotherapeutic targets in MM

To verify the effectiveness of our multi-cancer optimization analysis parameters on phenotypes that were not initially included in ImmunoTar’s optimization process and on data derived from adult samples, we utilized a surface proteomics dataset from adult-derived MM cell lines developed by Ferguson and colleagues (Ferguson *et al.* 2022). Within their work, the group developed a manual ranking system where they subjectively assigned points to each criterion extracted from their experiment data and four public databases to score their candidates. The approach identified C-C motif Chemokine Receptor 10 (CCR10) as a novel candidate immunotherapeutic target (Ferguson *et al.* 2022). Preliminary testing of this target showed that CCR10 could be a promising target for MM with some healthy tissue expression limitations (Ferguson *et al.* 2022). They also highly ranked a second target, Thioredoxin Domain Containing 11 (TXNDC11), noting that MM cells depend on TXNDC11 for proliferation in DepMap (Dwane *et al.* 2021). Their validation of TXNDC11 revealed that the protein was highly expressed in MM cell lines; however, it showed primarily intracellular localization and was considered a false-positive of their scoring system (Ferguson *et al.* 2022).

**Figure 4.**
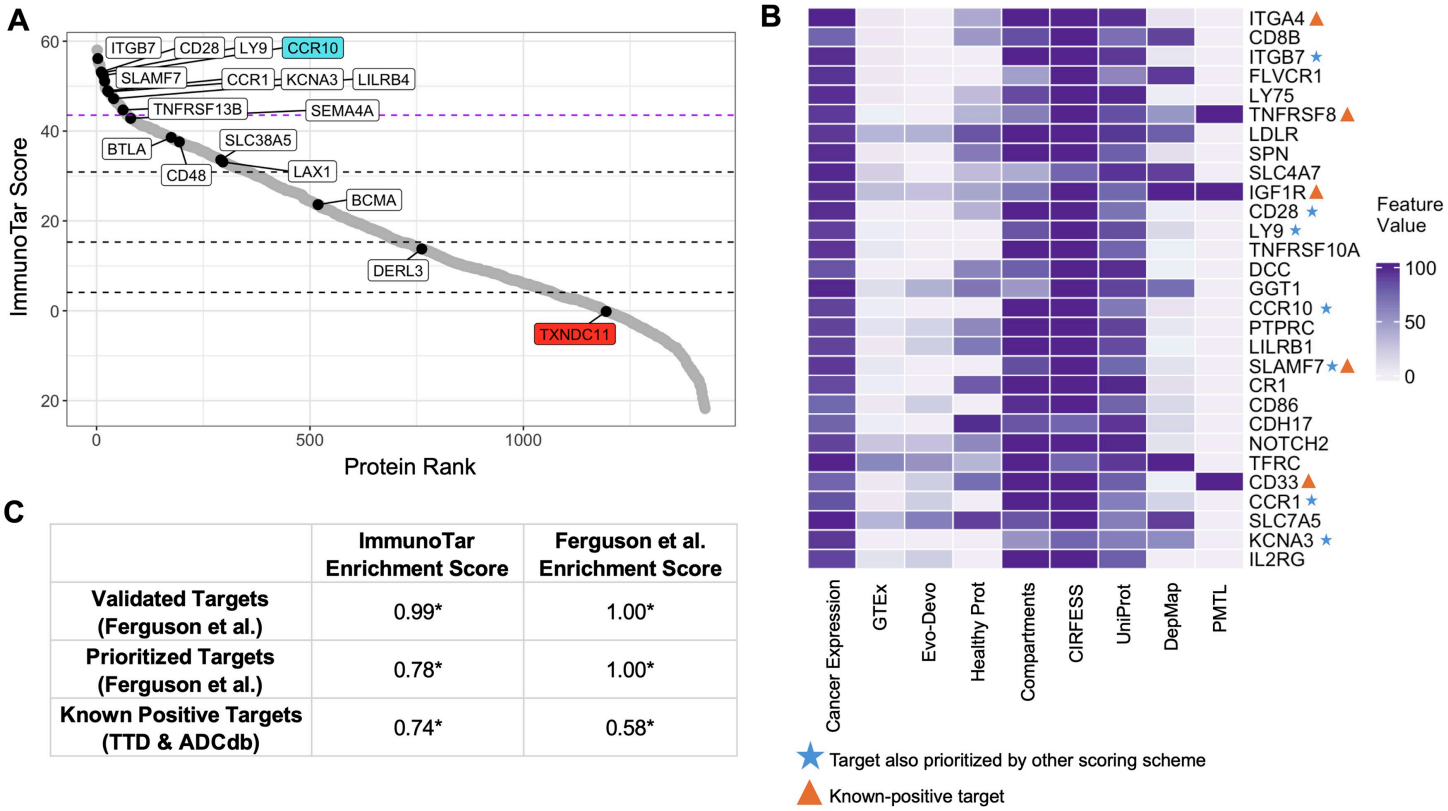
Validation of ImmunoTar using MM surface-proteomics data. (A) ImmunoTar results from the MM surface proteomics dataset using the multi-cancer optimization parameters. Labeled proteins are ones that were ranked in the top three scoring brackets by Ferguson and colleagues. The dashed line represents the top 5% scoring targets. CCR10, at the top of the curve, was prioritized and validated by Ferguson *et al.* as a novel target for MM. TXNDC11, at the bottom of the curve, is a prioritized protein that was a false-positive proved by functional validation. (B) The top 5% scoring targets using ImmunoTar, marking targets prioritized in ImmunoTar and the top three scoring brackets and targets that are known-positive targets per TTD and ADCdb. (C) The GSEA enrichment scores comparing three sets of prioritized targets in MM. The ImmunoTar scores of each target were compared to the target scores from the Ferguson *et al.* scoring scheme. *P-value ≤.05.

Analyzing the MM surface proteomics data with ImmunoTar using the multi-cancer optimization parameters, CCR10 scored in the top 20 targets while TXNDC11 scored in the lower score quantiles, consistent with it representing a false positive in Ferguson *et al.* (Fig. 4A, Supplementary Table S4). Completing a GSEA showed that ImmunoTar had a higher enrichment score in comparison to their scoring algorithm when looking at known-positive targets from TTD and ADCdb (Fig. 4B). Analyzing the proteins that scored in the top second percentile, 13 out of 29 (44%) were identified in the literature as being relevant to MM, either as biomarkers of the disease, involved in pathways implicated in MM, or related to disease risk (Fig. 4C). ImmunoTar ranked ITGA4 as the top scoring protein. ITGA4 is an adhesion molecule that is thought to enable multiple myeloma cells to stick to the bone marrow (Bou Zerdan *et al.* 2022). ITGA4 has previously been implicated in myeloma cell homing, survival, and acquisition of cell-adhesion mediated drug resistance (Nishinarita *et al.* 1998, Hosen 2020, Nair-Gupta *et al.* 2020, Hathi *et al.* 2022). Another target within the top-10 was Integrin Subunit Beta7 (ITGB7), an oncogenic adhesion molecule, also known to enhance MM cell adhesion to bone-marrow stroma, migration, and invasion (Neri *et al.* 2011, Roy Choudhury *et al.* 2023). Clinically, expression of *ITGB7* in MM is associated with poor survival outcomes post autologous stem cell transplantation (Neri *et al.* 2011). The GTEx expression of ITGB7 is limited to expression in the blood. Feline Leukemia virus subgroup C receptor 1, FLVCR1, also in the top 10 scoring proteins, is a membrane heme exporter protein. A study looking at MM gene dependencies showed that FLVCR1 was associated with MM cell survival and it has been reported to be possibly responsible for the poor prognosis associated with 1q32 gain in MM patients (Went *et al.* 2022).

### 3.3 ImmunoTar validates known EwS immunotherapy targets and highlights CADM1 as a new potential target

To test our optimization methodology and tool, we analyzed an EwS surface proteomics dataset from 19 pediatric EwS patient-derived or cell line-derived xenograft models (Mooney *et al.* 2023). In the original publication, Mooney and colleagues filtered their data to 218 proteins within the generated surface and global proteome datasets based on protein localization criteria (Mooney *et al.* 2023). They prioritized and scored this subset using a custom, in-house workflow that is not fully automated, assigning a ranking score to their data and three public databases. Through their system they identified and validated novel targets including Ectonucleotide pyrophosphatase/-phosphodiesterase 1 (ENPP1) and Cadherin 11 (CDH11) (Mooney *et al.* 2023).

**Figure 5.**
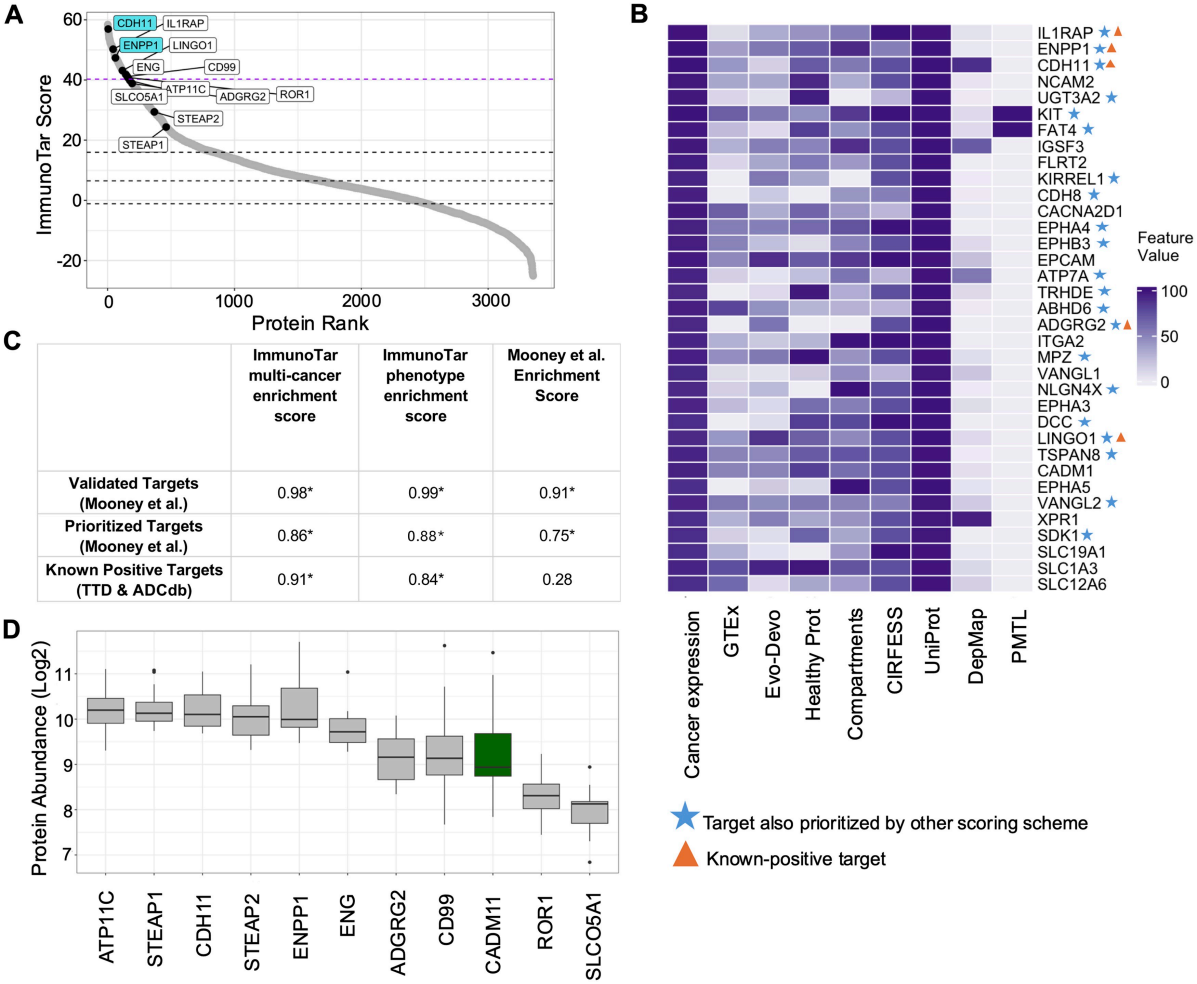
Validation of ImmunoTar using EwS surface proteomics data. (A) ImmunoTar results from the EwS surface proteomics data using the multi-cancer optimization parameter values. The dashed line represents the cut-off for the top 5% of proteins scored by ImmunoTar. The labeled proteins include targets that are known within EwS and highlighted by Mooney and colleagues. CDH11 and ENPP1, at the top of the curve, are novel targets discovered by Mooney and colleagues. (B) Heatmap showing the top 5% scored ImmunoTar targets after implementing a restricted normal-tissue expression filter. (C) The GSEA enrichment scores comparing three sets of prioritized targets in EwS. The ImmunoTar scores, using both the multi-cancer values and the phenotype-specific optimization parameter values, were compared to the original paper scores, separately. *P-value ≤.05. (D) Protein quantification of CADM1 in EwS surface proteomics dataset showing comparable abundance to other EwS-specific known-positive targets.

Using ImmunoTar, phenotype-specific optimized weight and curve values were generated with the cell surface proteins prioritized by Mooney and colleagues as known-positive targets. The multi-cancer and phenotype-specific optimization parameter values were each applied and ImmunoTar results from both were compared. Using the multi-cancer optimization approach, ENPP1 and CDH11, validated novel targets by Mooney and colleagues, scored in the top 2% of targets (Fig. 5A) and using the phenotype-specific prioritization parameters, IL1RAP, a previously validated target within EwS, ENPP1, and CDH11 scored as the top three targets (Fig. 5B; Supplementary Tables S5 and S6). To compare scoring approaches, we completed a GSEA using three different subsets of targets (Supplementary Table S7). Using both the multi-cancer and phenotype-specific optimization parameters, ImmunoTar provided a significantly high enrichment score of 0.86 and 0.88 (*P* <.0001) for the prioritized targets, respectively (Fig. 5C).

Filtering the output to highlight surface proteins with restricted normal tissue expression, defined as less than the 20th percentile expression across all tissues measured by GTEx, ImmunoTar identified 21 targets that were also prioritized by Mooney and colleagues in the top fifth scoring percentile (Fig. 5B; Supplementary Table S8). Notably, nine targets were identified within ImmunoTar that scored in the top scoring bracket, z-score >1, in the Mooney *et al.* scoring scheme including netrin receptor DDC (DCC), cadherin family member 14 (FAT4), and UDP-glucuronosyltransferase-3A2 (UGT3A2) (Fig. 5B).

A high scoring surface protein uniquely highlighted by ImmunoTar included cell adhesion molecule 1 (CADM1). CADM1 is a member of an immunoglobulin superfamily and has been involved in various tumor types such as ovarian cancer, breast cancer, and osteosarcoma (Lee *et al.* 2022, Wang *et al.* 2023). Studies suggest that CADM1 plays a role in in osteoblastic differentiation and bone formation, linking it biologically to bone cancers such as osteosarcoma and potentially Ewing sarcoma (Inoue *et al.* 2013, Wang *et al.* 2023). Protein expression of CADM1 in the surface-proteomics dataset is comparable to other know-positive targets highlighted by Mooney and colleagues in their work (Fig. 5D). Although CADM1 is expressed in some normal tissues (Supplementary Fig. S2), studies in osteosarcoma have reported minimal toxicity with a CADM1-targeted ADC in *in-vivo* studies (Wang *et al.* 2023). In small-cell lung cancer, researchers have identified a single-chain variable fragment specific to CADM1 in this phenotype and have developed an antibody therapy targeting it (Lee *et al.* 2022).

### 3.4 Phenotype-specific parameter values prioritize recently validated NBL immunotherapy target, DLK1

Leveraging our expertise in NBL, we applied ImmunoTar to a recently published PDX surface proteomics dataset that surveyed 12 pediatric NBL models (Hamilton *et al.* 2024). To demonstrate the application of the phenotype-specific parameters generated from one data source to a new dataset with available validation, we generated and utilized phenotype-specific optimization parameters using the NBL cell line full proteome data from multi-cancer dataset utilized above (Goncalves *et al.* 2022). To generate phenotype-specific parameters, we applied our optimization strategy using known-positive targets that have been explored in the literature or within our group including ALK, GPC2, CD276, L1CAM, and NCAM1 (Mosse *et al.* 2008, Rached *et al.* 2016, Bosse *et al.* 2017, Li *et al.* 2019, Tian *et al.* 2022, Rosswog *et al.* 2023, Hamilton *et al.* 2024).

**Figure 6.**
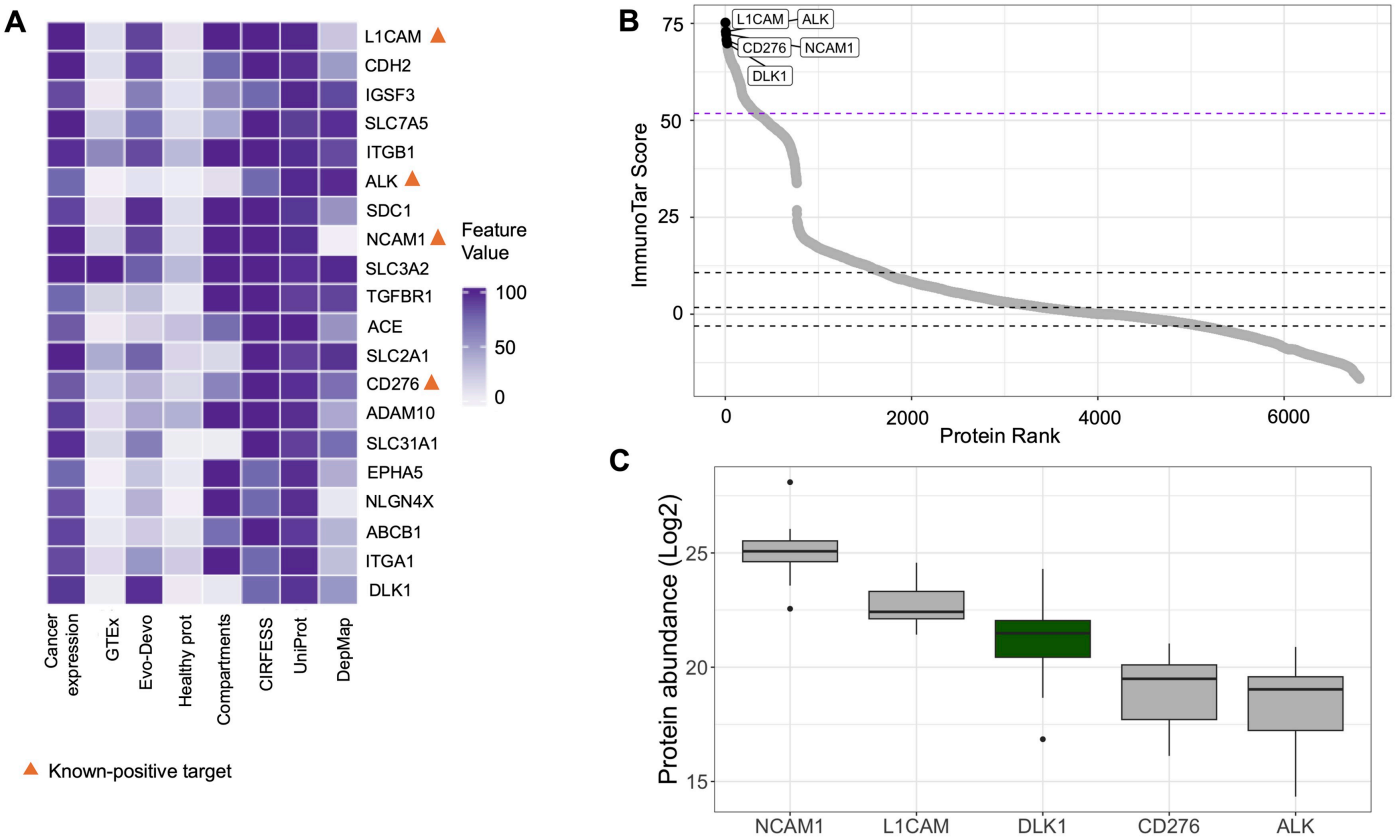
ImmunoTar prioritized targets in NBL using surface proteomics data from PDX models and phenotype-specific optimization parameters. (A) Evaluating the top 20 scoring targets per ImmunoTar analysis after applying phenotype-specific parameters. Highlighting known, top-scoring targets, L1CAM, ALK, NCAM1, and CD276. (B) Rank plot from ImmunoTar showing that DLK1 ranks in the top five percentile of scoring targets, comparable to other known targets. (C) Protein quantification of DLK1 in the surface proteomics data showing comparable abundance to other NBL-specific known targets.

Analyzing ImmunoTar’s results in the PDX surfaceome data showed that L1 cell adhesion molecule (L1CAM) was ranked as the top candidate in neuroblastoma, which is to be expected since it was utilized in the optimization algorithm as a known-positive target (Fig. 6A; Supplementary Table S9). Other known-positive targets, ALK, NCAM1, and CD276 also ranked among the top 20 scoring proteins. Notably, Delta-like canonical notch ligand 1 (DLK1), a novel target that has been recently validated by Hamilton et al, was ranked as the 20th top scoring protein (Fig. 6A and B) (Hamilton *et al.* 2024). DLK1 expression in the NBL surface proteomics data was comparable to other known targets (Fig. 6C). DLK1 is thought to contribute to the stem-like features and undifferentiated state of NBL cells (Begum *et al.* 2012). Hamilton *et al.* validated cell surface expression of DLK1 and showed that a DLK1-targeting ADC shows potent and specific cytotoxicity in NBL xenograft models expression DLK1 (Hamilton *et al.* 2024).

## 4 Discussion

We present ImmunoTar, a tool designed to prioritize potential cell surface proteins for cancer immunotherapy development. ImmunoTar analyzes user-provided cancer RNA and proteomic datasets, integrating these with generated quantitative features from several public databases including, GTEx, Evo-Devo, CIRFESS, COMPARTMENTS, UniProt, DepMap, and GO. This integration allows users to perform comprehensive quantitative evaluation of potential targets without individually accessing each database. Additionally, ImmunoTar includes drug databases like TTD, ADCdb, and PMTL, enabling users to identify existing therapies and reagents for the prioritized targets. This utility facilitates validation and translational research, highlighting ImmunoTar’s bench-to-bedside potential.

To provide optimized analysis parameter values for users, we used a pediatric multi-cancer cell line proteomics dataset and implemented both multi-cancer and phenotype-specific optimization strategies. Our analysis revealed that while phenotype-specific parameter values performed best on their respective phenotypes, multi-cancer parameter values offered a broader and more diverse applicability across different phenotypes. These findings suggests that users can utilize the multi-cancer optimization parameter values for their analyses, especially for cancers with few or no known positive cell-surface protein target.

To further validate ImmunoTar and our multi-cancer optimization parameter values, we benchmarked it against other scoring approaches and analyzed new datasets not previously evaluated by our tool. While the compared scoring approaches were effective, they were not designed as readily accessible tools for the research community and lacked full automation and usability across various datasets. ImmunoTar successfully replicated the results of other groups. Notably, in MM, a cancer phenotype not utilized to generate the optimized analysis parameter values and derived from adult samples, ImmunoTar identified targets previously reported by other groups such as ITGA4, ITGB7, and FLVCR1, confirming the robustness of our methodology and the applicability of our multi-cancer parameter values highlighting that while targets across different cancers may vary, the underlying characteristics of effective targets are consistent. Additionally, ImmunoTar identified a novel potential target, CADM1, in EwS with applicable biology that could explain its presence in EwS. In NBL, ImmunoTar successfully identified a recently published immunotherapeutic target, DLK1, ranking it in the top 20 targets. A Phase 1 first-in-human DLK1-directed immunotherapy clinical trial to treat neuroendocrine neoplasms, including neuroblastoma, in adult patients is currently open (ClinicalTrials.gov: NCT06041516). Results from EwS and NBL further demonstrate ImmunoTar’s ability to discover new therapeutic avenues. Across these three datasets, ImmunoTar was able to effectively capture immunotherapeutic target characteristics through its optimization parameter values, making it a versatile tool applicable to both adult and pediatric datasets, across various cancer phenotypes.

While ImmunoTar has demonstrated promising results, it is designed as a prioritization tool to help researchers efficiently sift through large datasets and identify potential candidate targets. Validation of the top-scoring proteins remains a critical step in confirming these candidates as surface protein targets. Additionally, ImmunoTar relies on user-provided RNA or proteomics datasets, which can be affected by data sparsity or contamination, influencing protein quantification and target prioritization. For instance, researchers may sometimes need to use cell line models due to limited availability of patient samples, which may not fully represent protein expression in actual tumors (Gillet *et al.* 2013). These discrepancies underscore the importance of high-quality, comprehensive datasets for accurate target identification using ImmunoTar, as well as the necessity of validating findings on human samples.

Although our optimization strategy and MAP score provide a standardized method for determining feature weights and curves and a way to evaluate the algorithm, both have their limitations in their dependence on targets from the TTD and ADCdb databases. Some targets included in either database can still be undergoing development and may lack finalized efficacy and safety results, potentially leading to the prioritization of false positives and thus also affecting the MAP score. Relying on these targets could result in the inappropriate assignment of weight and curve values to features that do not truly reflect an “ideal” target. Conversely, ranking discontinued targets as “known-negative” may not always accurately represent their potential, as therapy failure could be due to factors unrelated to the target itself. These factors need to be considered when deriving data from these databases. To overcome this limitation, ImmunoTar allows the user to incorporate additional target information beyond what is available in the provided databases, as demonstrated with the NBL case study where a curated list of known targets was used for optimization.

In our analyses, we focus on using ImmunoTar to analyze surface proteomic data, as it provides a targeted quantification of surface proteins compared to RNA-sequencing and full-proteome data. Another promising technique is immunopeptidomics, a targeted mass-spectrometry approach that discovers noncanonical antigens, tumor antigens not derived from traditional protein-coding regions of the genome. These antigens can be tumor-specific and shared among patients, making them valuable for immunotherapy (Fritsche *et al.* 2018, Chong *et al.* 2022). As large-scale tumor and normal tissue immunopeptidomics become available, ImmunoTar could be extended to incorporate these datasets. Currently, some tools focus on identifying tumor-specific proteins through alternative splicing and cancer-specific exons (Pan *et al.* 2023, Shaw *et al.* 2024). To broaden the range of target selection, future iterations of ImmunoTar could analyze short-read and long-read RNA-sequencing data for tumor-specific splice isoforms, as well as mass-spectrometry data for tumor-specific glycosylated proteins (Kahles *et al.* 2018, Thomas *et al.* 2021, Li *et al.* 2024).

To increase the efficacy of immunotherapies and prevent cancer relapse and therapy resistance, researchers have explored designing immunotherapies that target multiple cancer-specific proteins (dual therapies) (Hegde *et al.* 2013, Feng *et al.* 2017, Zhang *et al.* 2019). Dual targets can be expressed in different cell subsets, ensuring the therapy attacks all tumor cell subsets, or have opposite expression profiles in normal tissue, thereby decreasing on-target off-tumor effects and reducing toxicity. While ImmunoTar currently includes the features necessary for identifying such targets, a function that enables the systematic identification of dual target pairs or other pipelines such as the ones described by Dannenfelse *et al.* could be incorporated thus facilitating the development of dual therapies (Dannenfelser *et al.* 2020, Hamieh *et al.* 2023). Further extensions of ImmunoTar could incorporate tumor single-cell RNA-sequencing profiling databases, such as the Single-cell Pediatric Cancer Atlas (scPCA) (Hawkins *et al.* 2024). Such advancements will further solidify ImmunoTar’s role in the efficient discovery of high-potential cancer immunotherapy targets.

## 5 Conclusion

ImmunoTar is a versatile tool for identifying potential cell surface proteins for cancer immunotherapy. By integrating user-provided RNA-sequencing and proteomics datasets with quantitative features from multiple public databases, ImmunoTar offers a comprehensive and quantitative evaluation to identify cancer-specific proteins. Our validation across both pediatric and adult cancer datasets, including MM, EwS, and NBL, demonstrates its robustness and broad applicability. Despite certain limitations related to data quality and optimization strategies, ImmunoTar’s ability to streamline the identification of promising targets underscores its potential to accelerate the development of effective cancer immunotherapies. Future enhancements, such as incorporating immunopeptidomics and dual-target identification, will further expand its utility, paving the way for novel therapeutic approaches in oncology.

## Supplementary Material

btaf060_Supplementary_Data

## Data Availability

Data utilized in this study are publicly available and can be accessed as follows: The pan-cancer proteomics map protein intensity data and meta data were downloaded from cellmodelpassports.sanger.ac.uk as referenced by the publication (PMID: 35839778) (Goncalves *et al.* 2022). The EwS surface proteomics data was downloaded from Supplementary Data S1 table and the scoring metric for this dataset was downloaded from Supplementary Data S3 (PMID: 37812652) (Mooney *et al.* 2023). The Multiple Myeloma surface proteomic data was downloaded from Supplementary Data S1 and the scoring metric for this dataset was downloaded from Supplementary Data S2 (PMID: 35840578) (Ferguson *et al.* 2022). The NBL surface proteomics data taken from Hamilton *et al.* (PRIDE: PXD047474) (Hamilton *et al.* 2024). Data from each of the databases/datasets used to enrich ImmunoTar was accessed and downloaded on the following dates: COMPARTMENTS and CIRFESS, December, 2022; UniProt, February, 2024; EvoDevo, January, 2024; GTEx data was the Analysis V8 release accessed on January, 2024; DepMap and GO, September, 2022; Therapeutic Target Database (TTD), December, 2023; Data for the healthy tissue proteomics was downloaded from Supplementary Table S2 (PMID: 32916130). Data for OpenTargets was downloaded on July, 2023 (https://moleculartargets.ccdi.cancer.gov/fda-pmtl). ImmunoTar code is freely available on GitHub: https://github.com/sacanlab/immunotar.
