# Supplementary material for: ImmunoTar—integrative prioritization of cell surface targets for cancer immunotherapy
Source: Bioinformatics. 2025 Feb 11;41(3):btaf060. doi: 10.1093/bioinformatics/btaf060 (PMC11904301; doi:10.1093/bioinformatics/btaf060)
Supplement: btaf060_Supplementary_Data [file btaf060_supplementary_data.zip › Supplementary_Methods.docx]

**Supplementary methods**

**Enrichment databases included in ImmunoTar**

*Healthy tissue expression*

An essential criterion for selecting a target is minimal to no expression in healthy tissues to mitigate on-target off-tumor effects. To quantitatively define this criterion, RNA-sequencing data from the Genotype-Tissue Expression (GTEx) database was incorporated [1]. The GTEx project conducted RNA-sequencing on 17,382 samples from 948 donors, encompassing 54 non-diseased tissue types [21]. The majority of GTEx samples are derived from donors aged 20 years and above. TPM-normalized tissue and sample-level RNA-sequencing data were retrieved from the GTEx portal. As GTEx is primarily comprised of adult tissue samples, RNA-sequencing data from pediatric tissues, obtained through the Evo-Devo Mammalian organs (Evo-Devo) project, was also included [2]. For both RNA-sequencing datasets, quantitative features were generated at both tissue and sample levels, resulting in a total of ten quantitative features per database. These features encompass the highest RNA expression value across tissues and individual samples, the count of tissue types and individual samples with expression > 10 TPM/RPKM, and a normalized maximum expression feature adjusted to the sample count annotated to a specific tissue type within each database. The threshold of 10 TPM/RPKM represents the 90th percentile cut-off, designating samples/tissues exceeding this value as high expressing for the protein. Given that certain immunotherapies do not penetrate the blood-brain barrier, we extracted summary statistics both with and without brain samples included. Acknowledging potential disparities between RNA expression and protein abundance, we incorporated proteomics data derived from 201 samples representing 32 distinct normal human tissues generated by Jiang et al. [3]. The processed protein quantification file at both the sample and tissue levels from the publication was queried. We extracted the same features from this database as the healthy tissue RNA-sequencing databases; however, the threshold for high-expression varied based on the 90^th^ percentile protein expression value.

*Protein localization*

While surface proteins play a crucial role as biological markers for diseases and therapeutic targets, their characterization is challenging due to their relatively low abundance, making it difficult to capture them using common sequencing or proteomics technologies [4]. To address this limitation, computational resources have emerged to aggregate and predict information, aiding researchers in defining protein localization. Among these databases, we have incorporated data from Compiled Interactive Resource for Extracellular and Surface Studies (CIRFESS) [4]. CIRFESS integrates various prediction strategies and annotations to assign a score to each protein based on the collected evidence supporting its classification as a surface protein, the confidence scores in CIRFESS range from 0-4. To broaden the list of surface proteins and leverage multiple protein localization databases, we also included data from the COMPARTMENTS database [5]. COMPARTMENTS utilizes available proteomics datasets and publications to assign scores to proteins based on the confidence that a protein is located on the plasma membrane, with confidence scores ranging from 0-5. In the ImmunoTar data matrix, the confidence score assigned to each protein, was included from both COMPARTMENTS and CIRFESS. Additionally, the length of the extracellular component of proteins was included as a feature in the ImmunoTar data matrix. This length information was obtained from the UniProt Knowledgebase, a database providing protein sequences along with functional details [6].

*Biological significance*

To gain deeper insights into the biological roles of protein targets in the queried cancer phenotype, gene dependency lists from the DepMap project were incorporated [7]. Within the DepMap project, a comprehensive genome-scale RNAi and CRISPR-Cas9 genetic perturbation screen was conducted to silence or knockout individual genes, identifying those genes that impact cell survival across 501 human cancer cell lines [7]. The DepMap database provides probability scores for genes in each of the surveyed cell lines. In the ImmunoTar data matrix, the quantitative feature included from DepMap is the probability score from the cell lines annotated to the cancer phenotype of interest. When there are multiple cell lines annotated to a single cancer phenotype, the user can decide if they want to average the probability across all cell lines or choose the maximum probability across all cell lines to populate the DepMap feature in the data matrix. Additionally, information from the Gene Ontology (GO) database was included to identify gene sets associated with pathways relevant to the cancer phenotype [8]. The quantitative feature for GO is binary, indicating whether the gene is present or not in the pathways of interest specified by the user.

*Reagent/therapeutic availability*

In the pursuit of identifying targets with established reagents specifically in pediatrics, the NCI relevant Pediatric Molecular Targets List (PMTL) was incorporated. This list aims to facilitate the development of safe and effective new drugs for pediatric cancer treatment (source: https://moleculartargets.ccdi.cancer.gov/mtp-pmtl-docs). The quantitative feature included for this database is binary, indicating whether the gene is present in the PMTL. To broaden the search for targets with developed reagents beyond pediatrics, the Therapeutic Target Database (TTD) and The Database of Antibody-drug Conjugates (ADCdb) were included. TTD provides information on drugs associated with diseases, the mode of action of developed drugs, and the stage of testing for these drugs [9]. ADCdb is a database dedicated to listing antibody-drug conjugates, a class of immunotherapeutic drugs commonly used in cancer, that are approved or are in development and their associated disease [10]. This information from TTD and ADCdb enables researchers to explore repurposing already developed drugs, if applicable. The feature extracted from both the databases is a score associated with the phase of development of the drug in the disease of interest. The defined scores for each development phase are listed in **Supplemental Table S11**.

**ImmunoTar feature extraction**

*User-input*

In its initial phase, ImmunoTar constructs a data matrix with the initial columns being summary features of the user-input cancer expression dataset described in **Fig. 1B**. Using the input expression dataset, ImmunoTar can calculate the number of samples exhibiting "low-expression" of a gene. The user has the flexibility to either set their own threshold of “low-expression” or apply the default, below the 20th percentile of expression. Similarly, ImmunoTar can summarize the number of samples with "high-expression," allowing the user to set a cut-off for “high-expression” or apply the default, above the 80th percentile of expression. Finally, ImmunoTar can provide a summary of the number of samples expressing the gene without any cut-offs for expression. This approach yields five features representing the cancer-expression data. While the default is to include all these features in the data matrix, users can choose to exclude some of these features. Additionally, users can introduce their own experimental data summary, such as performing a differential expression analysis between experimental groups. In this scenario, ImmunoTar utilizes all the columns in the user-provided dataset for the scoring matrix. Following the summarization of the cancer-expression dataset, the user has the option to rescale and handle missing values for the summarized data, employing methods detailed in **Fig. 1C**. If nothing is specified, the defaults for rescaling and missing values are applied to the dataset.

*Enrichment databases*

We enrich the scoring matrix by integrating features from several public databases, enhancing the robustness and comprehensiveness of ImmunoTar's prioritization. Users can choose specific databases for enrichment or default to enriching with all databases. For GO, DepMap, TTD, and ADCdb databases, additional user input for enrichment is required. To enrich with the GO dataset, users must list pathways of interest, allowing ImmunoTar to query related gene sets. For DepMap, TTD, and ADCdb enrichment, users need to feed in the cancer phenotype, enabling ImmunoTar to retrieve cancer-specific gene-dependency lists from the DepMap project and known targets for that cancer from TTD and ADCdb. Additional options for TTD and ADCdb enrichment include specifying the mode of action of the drug, limiting the search query to certain types of developed therapies. Mode-of-action categories can be found in **Supplemental Table S12**. Following enrichment, users can opt to rescale and fill in missing values for the enrichment features.

*Applying curves and weights*

To account for non-linearity of the feature values associated to scores, we included a curving factor that can be applied to each feature. The formula for curving the feature values is shown in **Fig. 1C**. This will allow the algorithm to better discriminate between values especially at the extreme ends of the scale and for the system to differentiate between subtle differences in the feature values. After applying curves, the user can also apply varying weights to each enrichment feature to highlight important features. The product sum of the curved feature value and weight for each gene is then calculated, generating the final gene prioritization score.

**ImmunoTar evaluation and optimization**

*MAP score*

When extracting targets from TTD and ADCdb, some targets are associated with discontinued drug status and are given a negative score. Within ImmunoTar, targets that have a negative score are considered “known-negatives” and targets that have positive scores are considered “known-positives”. The MAP score for a phenotype that has both these types of targets is calculated as shown in **Fig. 2A**. The MAP score of the algorithm serves as a valuable metric for assessing the algorithm's performance as well as providing an opportunity for optimizing the scoring parameters.

*Optimization*

The aim of this optimization is to identify features within the ImmunoTar scoring data matrix that characterize known-positive targets. While the optimization can only be applied to phenotypes with known or associated targets, once the parameters are generated, they can be applied to phenotypes lacking known or associated targets. We incorporate several computational methods for optimization, including Sequential Feature Selection (SFS), Genetic Algorithm (GA), Nelder-Mead (NM), and Brent’s Method (BM). SFS iteratively adds features to a model to improve its performance, effectively handling high-dimensional data by ensuring the inclusion of the most informative features. GA utilizes principles of natural selection to explore large and complex search spaces, making it ideal for heuristic optimization [11]. NM algorithm, a simplex-based technique, is particularly useful for nonlinear optimization problems as it does not require gradient information [12]. Lastly, BM is a robust and efficient iterative technique for numerical root-finding, combining bisection, secant method, and inverse quadratic interpolation for faster convergence. The NM and BM methods are implemented using the optim library within R (<https://www.R-project.org>). The GA method was implemented using the GA R library [11].

*Gene-Set enrichment analysis (GSEA)*

GSEA was completed to compare how gene-sets scored in different scoring algorithms. Enrichment scores were calculated using fsgea library in R with p-values computed within the package [13].

**Datasets explored using ImmunoTar**

*Proteomic pediatric multi-cancer dataset and neuroblastoma (NBL) full proteome*

Our overall application of ImmunoTar in our work is summarized in **Fig. 2C**. Initially, we applied ImmunoTar using a proteomics dataset that surveyed 949 cancer cell lines across 28 tissue types, encompassing approximately 40 histologically diverse cancer types [3]. This dataset serves as a comprehensive proteomics resource, offering the opportunity to investigate proteomic profiles across a wide spectrum of cancer phenotypes. Gonçalves *et. al.* conducted mass spectrometry, generating full proteome data for each cell line. To ensure result consistency, three sets of technical duplicates were run for each cell line. In total, 8,498 proteins were quantified across the dataset, with a median of 5,237 proteins quantified per cell line.

Given our specific focus on pediatric cancers, we refined the dataset using available meta-data to include only pediatric cancer cell lines that each had at least three representative cell lines. The processed data, averaged across technical replicates and provided through supplementary files in their publication, served as the input for ImmunoTar**.** For our neuroblastoma (NBL) specific analysis, the NBL full proteome data from this dataset was utilized. The NBL data surveyed 22 pediatric NBL cell lines.

*Pediatric Ewing sarcoma (EwS) surface proteomics*

To validate the efficacy of our pipeline and optimized parameters and to compare our prioritization algorithm to ones used by other groups, we utilized a more focused surface proteomics dataset recently published by Mooney et al. [14]. This dataset was derived from tissue samples obtained from pediatric ES patient-derived or cell line-derived xenograft models [14]. A total of 19 samples were analyzed in technical or biological duplicates, depending on sample availability. Across all samples in the surface proteomics dataset, a total of 3,357 proteins were quantified. Within the publication, the team curated a list of 218 proteins that they annotated to surface proteins based on protein localization databases SurfaceGenie and UniProt. Next, they implemented a custom scoring methodology that combines data from their generated EwS specific surface and global proteome data, EwS RNA-sequencing data from the Pediatric Preclinical Testing Consortium (PPTC) and GTEx database to score the 218 proteins. This scoring methodology is not available as a tool to use for the research community. They identified novel potential candidates using this scoring mechanism that they proceeded to validate. For our methods, the processed proteomics data for each sample that was generated by the group and provided through the supplementary files of their publication was the input to ImmunoTar.

*Multiple Myeloma (MM) surface proteomics*

As a secondary analysis contributing to a cross-validation of our pipeline and optimized parameters in diverse experimental contexts and datasets, we applied our ImmunoTar pipeline to a surface proteomics dataset generated from MM cell lines by Ferguson et al. [15]. This dataset was derived from four multiple myeloma cell lines. The group ran technical duplicate or triplicate samples for each cell line for consistency of results. In total, 1,245 proteins were quantified in the surface proteomics cell line data. After quantification, the proteomics data was integrated with publicly available datasets including: RNA-sequencing data for MM cell lines from the Cancer Cell Line Encyclopedia (CCLE), RNA-sequencing data for blood cells from the Human Blood Atlas, and RNA-sequencing data for normal tissue from GTEx. For surface protein localization they included the Compartments database. They extracted 5 quantitative features from these databases related to surface abundance and specificity for plasma cells to identify immunotherapeutic targets for MM [15]. For their scoring algorithm, they included all genes identified in the surface proteomics data and additionally genes identified in MM bulk RNA-sequencing data, thus evaluating around 33,000 candidates. Using their scoring algorithm, they identified novel MM specific therapeutic candidates that they moved forward to validate in the lab. For our methods, we utilized the quantified proteomics files from the supplementary data and ran it through ImmunoTar.

**References**

1. Consortium, G.T., *The Genotype-Tissue Expression (GTEx) project.* Nat Genet, 2013. **45**(6): p. 580-5.

2. Cardoso-Moreira, M., et al., *Gene expression across mammalian organ development.* Nature, 2019. **571**(7766): p. 505-509.

3. Jiang, L., et al., *A Quantitative Proteome Map of the Human Body.* Cell, 2020. **183**(1): p. 269-283 e19.

4. Waas, M., J. Littrell, and R.L. Gundry, *CIRFESS: An Interactive Resource for Querying the Set of Theoretically Detectable Peptides for Cell Surface and Extracellular Enrichment Proteomic Studies.* J Am Soc Mass Spectrom, 2020. **31**(7): p. 1389-1397.

5. Binder, J.X., et al., *COMPARTMENTS: unification and visualization of protein subcellular localization evidence.* Database (Oxford), 2014. **2014**: p. bau012.

6. UniProt, C., *UniProt: the Universal Protein Knowledgebase in 2023.* Nucleic Acids Res, 2023. **51**(D1): p. D523-D531.

7. Dwane, L., et al., *Project Score database: a resource for investigating cancer cell dependencies and prioritizing therapeutic targets.* Nucleic Acids Res, 2021. **49**(D1): p. D1365-D1372.

8. Ashburner, M., et al., *Gene ontology: tool for the unification of biology. The Gene Ontology Consortium.* Nat Genet, 2000. **25**(1): p. 25-9.

9. Zhou, Y., et al., *Therapeutic target database update 2022: facilitating drug discovery with enriched comparative data of targeted agents.* Nucleic Acids Res, 2022. **50**(D1): p. D1398-D1407.

10. Shen, L., et al., *ADCdb: the database of antibody-drug conjugates.* Nucleic Acids Res, 2024. **52**(D1): p. D1097-D1109.

11. Scrucca, L., *GA: A Package for Genetic Algorithms in R.* Journal of Statistical Software, 2013. **53**(4): p. 1 - 37.

12. Nelder, J.A. and R. Mead, *A Simplex Method for Function Minimization.* The Computer Journal, 1965. **7**(4): p. 308-313.

13. Korotkevich, G., V. Sukhov, and A. Sergushichev, *Fast gene set enrichment analysis.* bioRxiv, 2019: p. 060012.

14. Mooney, B., et al., *Surface and global proteome analyses identify ENPP1 and other surface proteins as actionable immunotherapeutic targets in Ewing sarcoma.* Clin Cancer Res, 2023.

15. Ferguson, I.D., et al., *The surfaceome of multiple myeloma cells suggests potential immunotherapeutic strategies and protein markers of drug resistance.* Nat Commun, 2022. **13**(1): p. 4121.
