# Supplementary figures and images for: ImmunoTar—integrative prioritization of cell surface targets for cancer immunotherapy
Source: Bioinformatics. 2025 Feb 11;41(3):btaf060. doi: 10.1093/bioinformatics/btaf060 (PMC11904301; doi:10.1093/bioinformatics/btaf060)

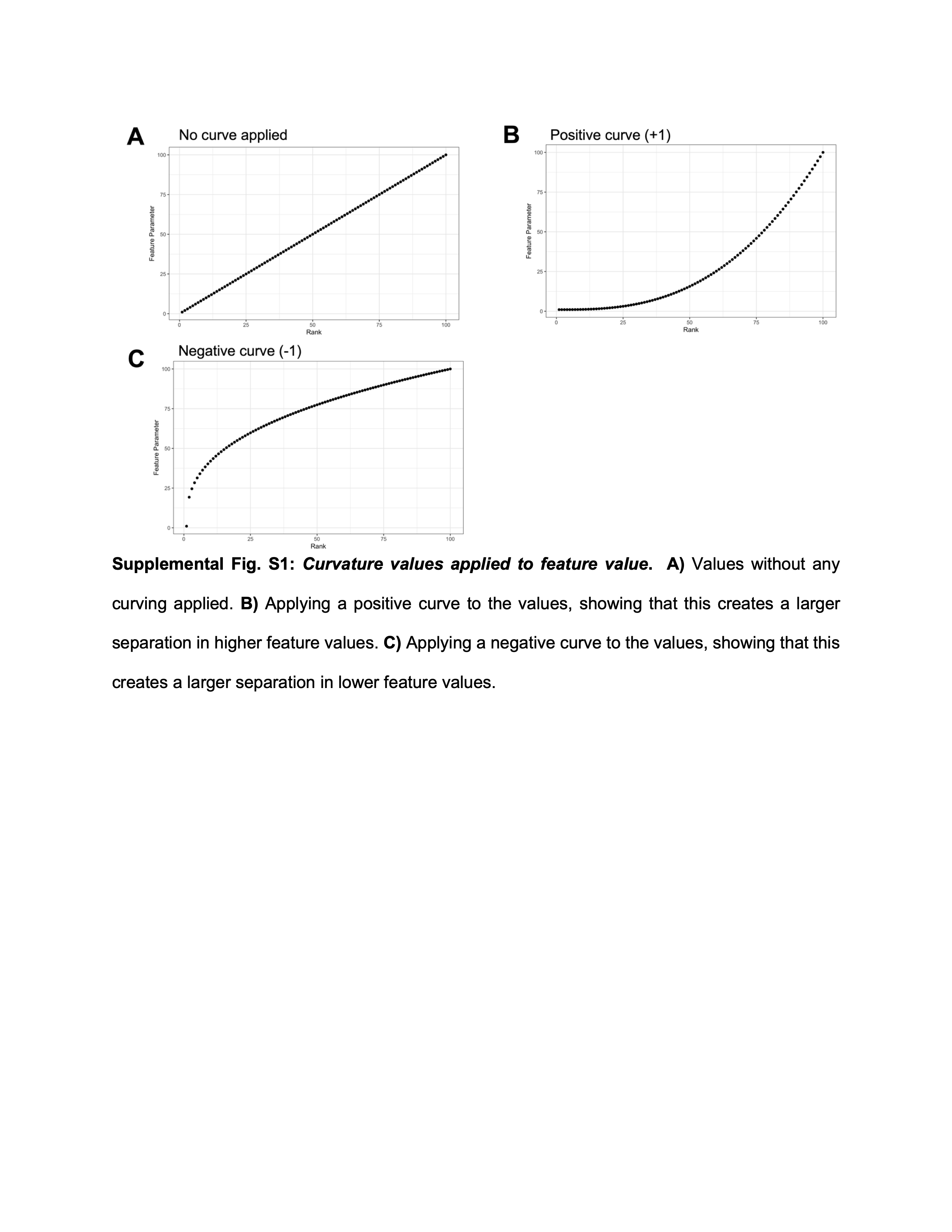

Supplement: btaf060_Supplementary_Data [file btaf060_supplementary_data.zip › Supplmental_Figs_S1-S2.png]
